# Detection of low-level parental somatic mosaicism for clinically relevant SNVs and indels identified in a large exome sequencing dataset

**DOI:** 10.1186/s40246-021-00369-6

**Published:** 2021-12-20

**Authors:** Daniel D. Domogala, Tomasz Gambin, Roni Zemet, Chung Wah Wu, Katharina V. Schulze, Yaping Yang, Theresa A. Wilson, Ido Machol, Pengfei Liu, Paweł Stankiewicz

**Affiliations:** 1 Department of Molecular and Human Genetics, Baylor College of Medicine, One Baylor Plaza, Houston, TX 77030 USA; 2 Graduate Program in Diagnostic Genetics, School of Health Professions, University of Texas at MD Anderson, Houston, TX USA; 3 Institute of Computer Science, Warsaw University of Technology, Warsaw, Poland; 4 Baylor Genetics, Houston, TX USA; 5 AiLife Diagnostics, 1920 Country Place Pkwy Suite 100, Pearland, TX USA

**Keywords:** Clinical diagnostic testing, Mosaicism carrier, Recurrence risk, Rare variants, Mendelian genomics

## Abstract

**Background:**

Due to the limitations of the current routine diagnostic methods, low-level somatic mosaicism with variant allele fraction (VAF) < 10% is often undetected in clinical settings. To date, only a few studies have attempted to analyze tissue distribution of low-level parental mosaicism in a large clinical exome sequencing (ES) cohort.

**Methods:**

Using a customized bioinformatics pipeline, we analyzed apparent de novo single-nucleotide variants or indels identified in the affected probands in ES trio data at Baylor Genetics clinical laboratories. Clinically relevant variants with VAFs between 30 and 70% in probands and lower than 10% in one parent were studied. DNA samples extracted from saliva, buccal cells, redrawn peripheral blood, urine, hair follicles, and nail, representing all three germ layers, were tested using PCR amplicon next-generation sequencing (amplicon NGS) and droplet digital PCR (ddPCR).

**Results:**

In a cohort of 592 clinical ES trios, we found 61 trios, each with one parent suspected of low-level mosaicism. In 21 parents, the variants were validated using amplicon NGS and seven of them by ddPCR in peripheral blood DNA samples. The parental VAFs in blood samples varied between 0.08 and 9%. The distribution of VAFs in additional tissues ranged from 0.03% in hair follicles to 9% in re-drawn peripheral blood.

**Conclusions:**

Our study illustrates the importance of analyzing ES data using sensitive computational and molecular methods for low-level parental somatic mosaicism for clinically relevant variants previously diagnosed in routine clinical diagnostics as apparent de novo.

**Supplementary Information:**

The online version contains supplementary material available at 10.1186/s40246-021-00369-6.

## Introduction

Somatic mosaicism occurs when a fraction of cells in the body contain a variant that arose as a result of a postzygotic mutation. For every cell mitotic division in an individual’s life, one to three mutations occur; however, by using the germline [1], few of these mutations are passed on to an offspring. If a mutation arises within the first eight cell divisions of human embryonic development, it can be present in both somatic cells and in the germline. Somatic mosaicism has been described in a wide variety of genetic disorders with all modes of inheritance and has been observed across different tissues [2–16]. Recent studies have shown that low-level somatic mosaic variants leading to neurodevelopmental disorders, congenital heart disease, and autism are more prevalent than previously thought [17–23].

It has been observed in parental blood samples that low-level (< 10%) somatic mosaicism may significantly increase the risk of passing pathogenic single-nucleotide variants (SNVs) or copy number variants (CNVs) to the offspring [24]. Variants present at low levels in an individual’s cells can be challenging to detect by standard clinical techniques, e.g., chromosomal microarray analysis (CMA), exome sequencing (ES), or Sanger sequencing. Such variants are often interpreted as de novo in the affected offspring when not detected routinely in the parental samples [25]. Additionally, the majority of somatic mosaicism studies have primarily analyzed DNA extracted from whole blood samples without sampling other tissues, including germline. Of note, the levels of mosaicism may vary greatly in whole blood as a result of somatic clonal expansion, especially in subjects with advanced age [26]. Furthermore, recent work has shown that blood cell lineages branch off from other cell lineages early in embryogenesis and that certain bottlenecks in embryonic development lead to heterogeneity in genetic mosaicism in different regions of the brain [27, 28]. Thus, it has been suggested that somatic mosaicism may not be measured in the most accurate fashion through sampling whole blood samples.

Recently, we have shown that variant allele fraction (VAF) in non-blood tissues such as the hair follicles, fibroblast, sputum, and buccal cells had higher VAF values than those in whole blood samples [29]. However, the number of analyzed samples was too low for statistical significance.

Here, we have queried the ES database at Baylor Genetics (BG) clinical genetics laboratory to identify parental low-level somatic mosaicism for clinically relevant single-nucleotide variants and small indels. ES data represent a highly enriched subset of clinically relevant variants that cause Mendelian disorders [30]. Sensitive molecular techniques were utilized to assess VAFs across different tissues in parents whose children have heterozygous or hemizygous clinically relevant SNVs or indels. The variation in VAFs across different somatic tissues and the difference between molecular techniques are discussed.

## Material and methods

### Sample collection

From each of the ten parents enrolled in the study, peripheral blood in EDTA tubes, buccal swab, urine, saliva, hair, and nail samples were collected (Additional file 1: Table S1). At home, blood draws and sample collection and shipping were provided by ExamOne (Quest Diagnostics, Secaucus, NJ).

### In silico analyses

**Fig. 1:**
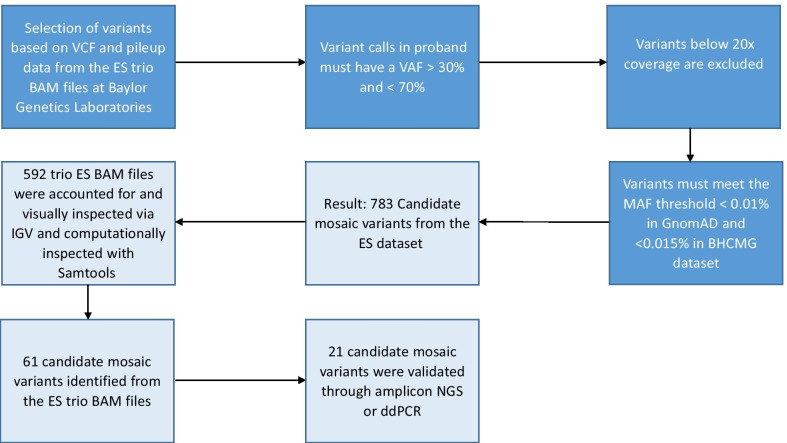
Filtering for mosaic variants using a bioinformatics pipeline. VCF files were analyzed to find variants that were heterozygous in probands. Variants that were called as heterozygous with a VAF < 30% or above 70% were eliminated. Variants with a read coverage below 20 × were excluded. It was also required that variants had a MAF < 0.01% in GnomAD and < 0.015% in the BHCMG dataset and were not located in repetitive gene regions or pseudogenes. Variants with VAF below 10% mosaic were not called using standard mosaic variant calling pipelines

A custom bioinformatics script described by Gambin et al. [29] was used to index the ES database at BG laboratories to select trios (DNA extracted from peripheral blood) where the proband had an apparent de novo and clinically relevant heterozygous or hemizygous SNV or small indel (Fig. 1). To select candidates variant calls in probands, erroneously called as heterozygous with a VAF > 70% and < 30% and variants with ES read depth coverage below 20X were excluded. Criteria for candidate variants also included a MAF below 0.01% in GnomAD (v2.1) and < 0.015% in the Baylor Hopkins Center for Mendelian Genomics (BHCMG) dataset. Variants located in repetitive and pseudogene genomic regions were filtered out. The analyses of the pileup data were performed using Samtools version 1.13 [31].

### Molecular analyses

Following Sanger sequencing, validation of the putative parental somatic mosaic variants was performed using PCR amplicon-based next-generation sequencing (amplicon-NGS) (Cloudhealth Genomics, Shanghai, China) and droplet digital PCR (ddPCR). The experimental workflow to assess low-level somatic mosaic clinically relevant SNVs and small indels was largely based protocols developed by Liu et al. [32] for detection of somatic mosaic CNV deletions.

#### DNA extraction

DNA from peripheral blood was extracted using the Gentra Puregene Blood Kit (Qiagen, Germantown, MD, USA). The QIAamp DNA Investigator Kit (Qiagen) was used to extract DNA from at least five hair follicles and nail clippings from fingers or toes. The ORAcollect OGR-500 kit (DNA Genotek, Ottawa, Canada) and the ORAcollect OC-175 kit (DNA Genotek) were used to collect saliva and buccal cells, respectively. The prepIT-L2P (DNA Genotek) DNA extraction reagent was used to extract DNA from buccal cells and saliva. Urine was extracted within 24 h after collection using the Quick-DNA Urine Kit (Zymo Research, Irvine, CA, USA). All procedures followed the manufacturer’s protocols.

#### Sanger sequencing

Sanger sequencing was performed in probands where low-level mosaicism was suspected in a parent to ensure that proband and parental samples were not misidentified. In addition, Sanger sequencing was used as a validation of the primers created for use in for amplicon NGS sequencing to determine whether the variant of interest could be observed in the proband. If the variant of interest was observed in the middle of the Sanger sequencing output in both forward and reverse sequences in the proband sample, parental amplicons containing the region of interest were sequenced via PCR amplicon NGS.

#### Amplicon NGS

The putative mosaic variants were targeted by PCR primers designed using the Primer3Plus tool. Candidate mosaic parental samples were amplified by PCR using Dreamtaq DNA polymerase (Thermofisher Scientific, Waltham, MA, USA). PCR products for each parental sample were purified using the QIAquick PCR Purification Kit (Qiagen) following the manufacturer’s protocol. The concentration of the PCR products was quantified using the Qubit dsDNA BR Assay (ThermoFisher Scientific) by the Qubit 4 Flourometer (ThermoFisher Scientific). Purified PCR products of 150–280 bp in length were sequenced using the Illumina Novaseq platform (Illumina, San Diego, CA, USA) with 150-bp paired end reads at Cloud Health Genomics (Shanghai, China). Data were analyzed using BWA-mem, Integrative Genomics Viewer (IGV), and GATK HaplotypeCaller for putative mosaic indels. Custom scripts were written in R statistical programming language.

#### ddPCR

Droplet digital PCR primers and FAM and HEX probes were designed by and purchased from Integrative DNA Technologies (IDT) (Coralville, IA, USA). Reactions were prepared in 20 µl aliquots, with 10 µl of ddPCR supermix for probes (no dUTP), 0.5 µM forward and reverse primer, 4 units of *Hind*III-HF (New England Biolabs, Ipswich, MA, USA), and 25 ng of DNA added. In every experiment, DNA from the proband’s blood sample was used as a positive control, and unrelated wildtype DNA from an unrelated blood sample was used as a negative control. Dilution series of the variant of interest were performed using gBlocks (synthetically generated double-stranded DNA sequences that ranged in size from 250 to 500 bp in length) (IDT) to determine the lowest level of detection for mosaic variants. These were subsequently used as positive and negative controls for validation of ddPCR assays, in SNV and small indel ddPCR assays. To test for contamination, a no-template control was used when testing each parental mosaic candidate.

## Results

### Querying the BG ES database using a custom bioinformatics pipeline

**Fig. 2:**
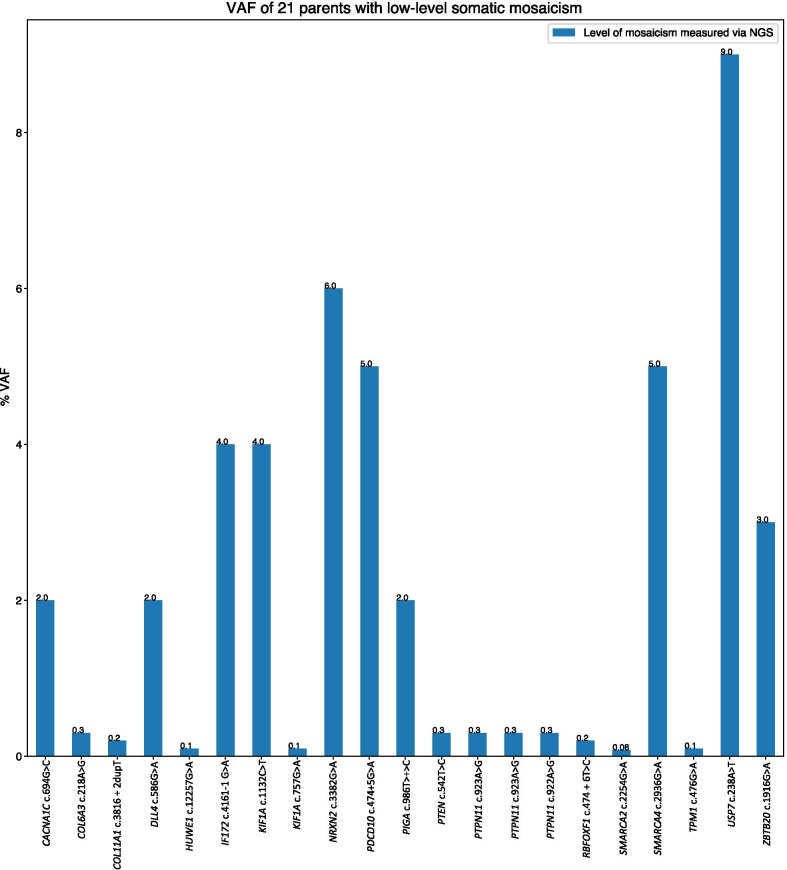
VAF distribution among 21 parents that tested positive for low-level somatic mosaicism using amplicon NGS

We initially selected 783 trios from the ES database at BG. For 191 (24%) trios, the BAM files could not be retrieved for either parents or probands (Fig. 1). As a result, the BAM files of 592 trios were queried for parental low-level somatic mosaicism using a custom bioinformatics pipeline (Fig. 1). Sixty-one (10.3%) individuals were selected using computational methods based on the *FracSampleswithAlt* cutoff value of < 10% in the parental ES samples and visually screened using the IGV (Additional file 1: Fig. S1). Of the 61 mosaic candidates verified using computational methods, 21 (21/592, 3.5%) tested positive for low-level mosaicism using amplicon NGS (Table 1, Figs. 1, 2, Additional file 1: Table S2, Fig. S1). In these 21 DNA samples, the ratio of the clinically relevant variants had a paternal to maternal ratio of 9:12 (Additional file 1: Fig. S2).

**Table 1:** Characterization of 21 parental somatic mosaic variants identified in peripheral blood using clinical ES at BG

Gene	Variant	ACMG variant classification	Proband phenotype	Inheritance mode	Parental mosaicism	Level of mosaicism in ES (%)	Level of mosaicism in NGS (%)	Level of mosaicism in ddPCR
*CACNA1C*	c.694G > C	VUS	Timothy Syndrome [MIM:601005]	AD	Maternal	1	2	NA
*COL6A3*	c.218A > G	VUS	Bethlem myopathy [MIM:58810]	AD/AR	Maternal	0.4	0.3	NA
*COL11A1*	c.3816 + 2dupT	Pathogenic	Stickler Syndrome 2: [MIM:604841]	AD/AR	Maternal	2.8	0.2 (HC)	NA
*DLL4*	c.586G > A	Likely pathogenic	Adams-Oliver syndrome [MIM:616589]	AD	Paternal	2	2	0.05%
*HUWE1*	c.12257G > A	Likely pathogenic	Mental retardation, X-linked, turner type [MIM:30076]	XL	Maternal	0.3	0.1	NA
*IFT172*	c.4161-1G > A	Pathogenic	Retinitis pigmentosa [MIM:616394]	AR	Paternal	3	4	3.8%
*KIF1A*	c.1132C > T	VUS	Polyhydramnios	AD/AR	Maternal	2	4	3.7%
*KIF1A*	c.757G > A	Pathogenic	Mental retardation, autosomal dominant 9 [MIM:614255]	AD/AR	Paternal	1	0.1	NA
*NRXN2*	c.3382G > A	VUS	Autism spectrum disorder	AD	Maternal	5	6	4.5%
*PDCD10*	c.474 + 5G > A	Pathogenic	Cerebral cavernous malformations 3 [MIM:603285]	AD	Paternal	1	5	NA
*PIGA*	c.986T > C	Likely pathogenic	Multiple congenital anomalies-hypotonia-seizures syndrome 2 [MIM:300868]	XLR	Maternal	2	2	1.83%
*PTEN*	c.542T > C	Pathogenic	Cowden syndrome 1 [MIM:158350]	AD	Maternal	2	0.3	NA
*PTPN11*	c.923A > G	Pathogenic	Noonan syndrome 1 [MIM:163950]	AD	Maternal	1	0.3	NA
*PTPN11*	c.923A > G	Pathogenic	Noonan syndrome 1 [MIM:163950]	AD	Maternal	1	0.3	NA
*PTPN11*	c.922A > G	Pathogenic	Noonan syndrome 1 [MIM:163950]	AD	Maternal	1	0.3	NA
*RBFOX1*	c.474 + 6T > C	VUS	Autism spectrum disorder	AD	Paternal	1	0.2	NA
*SMARCA2*	c.2254G > A	Likely pathogenic	Nicolaides-Baraister syndrome [MIM:601358]	AD	Paternal	1	0.08	NA
*SMARCA4*	c.2936G > A	Pathogenic	Coffin Siri syndrome 4 [MIM: 614609]	AD	Paternal	4	5	4.76%
*TPM1*	c.475G > A	Likely pathogenic	Cardiomyopathy [MIM:115196]	AD	Paternal	2	0.1	NA
*USP7*	c.238A > T	Likely pathogenic	Intellectual disability and autism spectrum disorder [PMID: 26365382]	AD	Maternal	3	9	4.13
*ZBTB20*	c.1916G > A	Likely pathogenic	Primrose syndrome [MIM:259050]	AD	Paternal	1	3	NA

*AD* autosomal dominant, *AR* autosomal recessive, *HC* GATK haplotype caller, *NA* none accountable, *VUS* variant of unknown significance, *XLR* X-linked recessive

### Molecular analyses

One parent was validated molecularly as being low-level mosaic for a clinically relevant variant that was previously diagnosed as apparent de novo in 3.4% (21/592) of trios. Out of ten families consisting of eight mothers and two fathers enrolled in the study, we have obtained different tissue samples from nine families, five of which tested molecularly positive for low-level mosaicism (Tables 2, 3).

#### Amplicon NGS

Twenty-one (34.4%) out of 61 blood samples with VAFs in ES data ranging from 0.3 to 5% (median 1.5%) tested positive for parental somatic mosaicism using amplicon NGS. VAFs ranged from 0.08% to 11.0% (median 0.3%) (Table 1). The average read depth at the variant position in question was 621,899×.

#### Droplet digital PCR validation

Dilution series were performed using gBlocks controls and artificial or mock low-level mosaics that established a lower level of detection of 0.5 VAF percentage to establish the limit of detection of low-level mosaic variants using ddPCR (Additional file 1: Fig. S3).

#### Level of mosaicism in peripheral blood

VAF percentages for low-level mosaic variants in blood tested using ddPCR ranged between 0.05 and 4.76% (median 3.7%) (Table 1). The highest VAFs of 4.76% for the c.2936G > A variant in *SMARCA4* and 4.1% for the c.3382G > A variant in the *NRXN2* gene were measured using ddPCR in peripheral blood. Accordingly, the highest VAFs measured by amplicon NGS were 5% for the c.2936G > A variant in *SMARCA4* and 6% for the c.3382G > A variant in the *NRXN2* gene. The c.1132C > T and c.4161-1G > A variants in *KIF1A* and *IFT172* had VAFs of 3.7% and 3.8%, respectively, when measured using ddPCR in the blood. The VAF percentage of 4.0% was observed for both variants when amplicon NGS was performed. The lower end of VAF measurements using ddPCR was observed for the c.986T > C and c.586G > A variants in *PIGA* and *DLL4* with VAFs of 1.8% and 0.05%, respectively. The VAFs measured using amplicon NGS were 2.0% for both *PIGA* c.986T > C and *DLL4* c.586G > A variants.

Of note, 3 out of 21 (14%) unrelated parents were found to be candidate somatic mosaic for the *PTPN11* pathogenic variants causative for Noonan syndrome, with an average VAF percentage measured by amplicon NGS of 0.3%.

The largest difference observed in the level of mosaicism over time in blood leukocytes was observed in parent M1 where the differences in VAF decreased by 2% (Additional file 1: Table S3). No other changes in VAF in DNA extracted from blood leukocytes were observed across other samples.

#### Comparison of low-level mosaicism across different somatic tissues

DNA extracted from blood leukocytes had the highest detected VAF compared with other tissues when mosaicism was assessed using amplicon NGS. The mean and median VAF differences between measurements taken using amplicon NGS and ddPCR were 2.99% and 2.18%, respectively. Mosaicism level measured by amplicon NGS in blood leukocytes ≤ 2.0% was not detected by ddPCR.

Of five families enrolled, low-level mosaicism was detected in all sampled tissues in only one mother (M1) with the c.238A > T likely pathogenic variant in the *USP7* gene through the use of amplicon NGS and ddPCR. Parent M1 with a VAF percentage measured by ddPCR in peripheral blood leukocytes of 2.13% had the highest VAF measured by ddPCR in the saliva (4.78%) and the lowest VAF in a buccal swab sample (0.3%) (Additional file 1: Fig. S4). The variance in the VAFs across tissues measured by amplicon NGS was 14.1%, the variance for M1 was 2.89%. In 60% (3/5) of parents, low-level mosaicism could be detected in peripheral blood leukocytes using amplicon NGS, but was undetectable by ddPCR. In subject M5, mosaicism was not detected in the blood using ddPCR, but was detected at a VAF of 0.2% in the saliva and urine samples.

#### Inheritance pattern

Notably, 71% (15/21) of low-level somatic mosaic variants were in autosomal dominant (AD) trait genes, 14% (3/21) in autosomal dominant/autosomal recessive disease (AD/AR) trait genes, 9% (2/21) in X-linked trait genes, and 4% (1/21) in AR trait genes (Additional file 1: Fig. S5).

## Discussion

Our recent study [29] of 102 parents with candidate mosaic variants validated using amplicon NGS, ddPCR, or blocker displacement amplification (BDA) [33] revealed 27 (26.4%) as low-level mosaic (VAF percentage between 1 and 10%) or very low-level mosaic (VAF percentage < 1%). Here, we have sought to expand the sample size of tissues from parents with suspected low-level mosaic clinically relevant SNVs or indels to determine whether whole peripheral blood is the optimal tissue to assess low-level parental somatic mosaicism. Using a customized bioinformatics pipeline, we have queried the ES database and found that approximately 3.4% of clinically relevant variants diagnosed as apparent de novo events are in fact low-level parental somatic mosaicism. This study is unique in that it is restricted to clinically relevant variants identified in a large ES dataset that meet the ACMG criteria of being pathogenic, likely pathogenic, or variant of unknown significance [34].

To date, most of the somatic mosaic variants that result in a single damaging event with a large phenotypic effect have been reported to be more common in neurodevelopmental disorders with an AD inheritance pattern [14]. Consistent with these findings, we have observed that neurodevelopmental disorders due to variants in AD trait genes, including cerebral cortical malformations, autism spectrum disorder [19], and epileptic encephalopathy [3], were in a large proportion of the study cohort (Additional file 1: Table S2). However, this apparent enrichment can be reflective of these phenotypes being primarily referred for trio ES testing at BG. Mosaicism in traits that are AR is rare and requires that a variant allele is inherited from one parent in addition to a de novo event occurring [35, 36].

There was no disproportionate difference between the number of clinically relevant low-level mosaic variants inherited paternally (42%) or maternally (57%). The observed ratio close to 1:1 has been observed in previous studies of somatic mosaicism [37] and contrasts with gonadal mosaicism which is skewed to paternal inheritance due to high number of divisions occurring during spermatogenesis [38].

In some disorders, it is necessary to sample for mosaicism in tissues other than blood. For example, in patients with Pallister–Killian syndrome, patch-like patterns occurring in skin may need to be sampled for tetrasomy of isochromosome 12p [39] due to mosaicism being limited at that site. In addition, clonal expansion of peripheral blood leukocytes may lead to an erroneous conclusion of an increased level of mosaicism over time. Therefore, using more sensitive and precise molecular techniques, we have measured variation in the level of mosaicism also across different somatic tissues. Analysis of low-level mosaic clinically relevant variants in five families revealed variation in VAFs across blood, buccal, saliva, urine, hair, and nails. Fluctuations in VAF percentages across all tissues samples were observed only in one mother (M1). The c.238A > T likely pathogenic variant in the *USP7* gene was present at the highest VAF in samples taken from the mesoderm germ layers (blood, saliva). Samples taken from tissues in the other germ layers were observed to have more variable VAFs, with an observable variation in the VAFs in samples taken from the hair and nails which represent the ectoderm germ layer. Hair and nails tissue samples had the most outlying VAFs, which has been observed previously [29].

Hair is comprised of 95% protein and yields a small amount of DNA template which could possibly lead to a variable assessment of somatic mosaicism [40]. Extraction of high quality genomic DNA from nails can be hindered when DNA is fragmented during the keratinization process that occurs during cellular growth [41].

In three parents with low-level mosaicism, the clinically relevant variant of interest was detected in urine. Urinary sediment can have trace amounts of leukocytes, erythrocytes, and urinary epithelial cells [42]. Assessment of the degree of variation in VAF for clinically relevant low-level mosaic variants across different tissues can be useful to clinicians to determine at what stage of embryogenesis the variant arose. This in turn may help to determine whether they might be present in germline and transmitted to progeny.

Use of NGS (at an average coverage depth of 621,899×) enabled the detection of mosaic variants with VAFs that would have been missed using standard clinical methods. Of note, sequencing at a depth of over 2000× read coverage does not provide additional information even in recent NGS platforms such as Illumina Novaseq. The error rate in amplicon NGS depends on several factors, e.g., DNA polymerase, NGS workflow used, sample handling, and the type of PCR enrichment performed, not allowing for substantial improvement in the sensitivity rate. Gambin et al. [29] observed that detection of VAFs in NGS below 1% could not be verified using ddPCR. ddPCR has been reported to have a theoretical VAF sensitivity rate of 0.001%, and our previous ddPCR experiments have been able to detect somatic mosaic variants in the *FOXF1* gene at a cutoff sensitivity VAF of 0.1% [43–45]. We have utilized ddPCR in seven parents. A discrepancy between these methods was observed in four cases. VAFs below 2% detected using NGS in peripheral blood leukocytes were not identified using ddPCR. Moreover, the same variant c.923A > G in *PTPN11,* identified as 0.3% mosaic in amplicon NGS studies in three unrelated parents (Table 1), but was not verified by ddPCR across different tissues in parents M2 and M3 (Tables 2, 3), indicating that it may represent a technical artifact. In parent M5, 0.2% mosaicism for the c.694G > C variant in *CACNA1C* was detected using ddPCR in the saliva and urine samples, but it was not found using amplicon NGS. These results illustrate the value of validation using multiple sensitive molecular techniques such as ddPCR and amplicon NGS for clinically relevant low-level mosaic variants. Additionally, these results provide further evidence for genetic counselors that sensitive molecular techniques such as PCR amplicon NGS or ddPCR may be used in detection of low-level mosaic clinically relevant variants that are diagnosed as apparent de novo.

**Table 2:** Ten parents suspected of low-level somatic mosaicism enrolled in this study

Parent	Gene	Variant	Mosaic using NGS	Tissues received
M1	*USP7*	c.238A > T	Yes	Yes
M2	*PTPN11*	c.923A > G	Yes	Yes
M3	*PTPN11*	c.923A > G	Yes	Yes
M4	*COL11A1*	c.3816 + 2dupT	Yes	Yes
M5	*CACNA1C*	c.694G > C	Yes	Yes
M6	*SYNGAP1*	c.1782dupC	No	Yes
M7	*SYNGAP1*	c.654_655delCT	No	Yes
M8	*PUF60*	c.628C > T	No	Yes
F1	*STXBP1*	c.1099C > T	No	No
F2	*NEDD4L*	c.814-6 T > A	No	Yes

*M* mother, *F* father

**Table 3:** Results displaying the VAF percentages of amplicon NGS and ddPCR analyses performed on different somatic tissue samples from parents suspected of low-level mosaicism

Parent	Gene	Variant	Blood			Buccal		Saliva		Urine		Hair	Nails
			ES (%)	PCR NGS (%)	ddPCR	PCR NGS	ddPCR	PCR NGS	ddPCR	PCR NGS	ddPCR	PCR NGS	PCR NGS
M1	*USP7*	c.238A > T	3	9	2.13%	1%	0.3%	8%	4.78%	2%	0.86%	0.03%	5%
M2	*PTPN11*	c.923A > G	1	0.3	ND	0.21%	ND	0.1%	NT	0.22%	NT	0.15%	NA
M3	*PTPN11*	c.923A > G	1	0.3	DEG	0.2%	ND	0.2%	NT	0.2%	NT	NA	NA
M4	*COL11A1*	c.3816 + 2dupT	2.8	0.2 (HC)	ND	ND	ND	ND	DEG	ND	ND	ND	ND
M5	*CACNA1C*	c.694G > C	1	2	ND	ND	ND	ND	0.2%	ND	0.2%	0.05%	ND

*HC* GATK haplotype caller, *NA* not accountable, *ND* not detectable, *NT* not tested, *DEG* degraded DNA

Recently, novel techniques for detecting and precisely measuring low-level somatic mosaicism even below 0.1% have been described. BDA can reliably detect VAFs even below 0.1% [46]. MIPP-Seq that utilizes unique molecular identifiers to increase assay sensitivity can be used for measuring VAFs for SNVs and indels as low as 0.025% [47]. The use of a low number of PCR cycles along with the use of multiple independent primers that cover the variant region leads to less allelic dropout. Methods such as these can be used as a means of orthogonal validation in addition to PCR amplicon NGS and ddPCR, to determine if a low-level mosaic variant with a VAF < 2% is an artifact. Molecular barcoding techniques such as MIPP-Seq could be utilized to validate low-level somatic mosaic variants including SNVs, CNVs, and indels. However, these methods can be cost prohibitive to implement.

The vast majority of the variants identified here as low-level mosaic were SNVs and only a few indels. This bias may result from low-level mosaic indels presenting a detection challenge both bioinformatically in analyzing NGS data and for designing FAM and HEX probes for ddPCR. Reads where indels occur are often filtered out during sequence alignment, which may lead to erroneous indel calling. Secondly, overlapping reads are more difficult to align and may consequently be mapped with incorporated mismatches [48]. In a mother (M5) with a *COL11A1* c.3816 + 2dupT pathogenic variant, the mosaic insertion could only be detected in the blood through use of GATK haplotype caller, and was not found in other tissues. This finding was unexpected as the insertion was observed in 2.8% of reads generated from ES. Previous studies have found that indels occurring in the human genome are missed at a rate of 10–35% [49]. However, the rate that low-level somatic mosaic indels present in the human genome could be missed is more than 35% of the time.

## Conclusions

The rate of mosaicism observed in the ES dataset (3.4%) corroborates the previous studies. The differences in VAF percentages among sampled non-blood tissues add to the notion that other tissues may be informative in detection and quantification of low-level somatic mosaicism. Low-level somatic mosaic indels are difficult to detect due to filtering and mapping challenges. Use of multiple sensitive molecular techniques in addition to assessing multiple tissues for clinically relevant variants should be considered by investigators and clinicians for validation of low-level parental somatic mosaicism. These practices would facilitate more accurate assessment of recurrence risk.

## Supplementary Information

**Additional file 1.** Supplementary Tables S1-S3 and Figures S1-S5.

## Data Availability

Please contact the author for data requests.

## Abbreviations

amplicon NGS: PCR amplicon next-generation sequencing
BCM: Baylor College of Medicine
BDA: Blocker displacement amplification
BG: Baylor genetics
BHCMG: Baylor Hopkins Center for Mendelian Genomics
CNV: Copy number variant
ddPCR: Droplet digital PCR
ES: Exome sequencing
SNV: Single-nucleotide variant
VAF: Variant allele fraction
