# Supplementary material for: Detection of low-level parental somatic mosaicism for clinically relevant SNVs and indels identified in a large exome sequencing dataset
Source: Hum Genomics. 2021 Dec 20;15:72. doi: 10.1186/s40246-021-00369-6 (PMC8686574; doi:10.1186/s40246-021-00369-6)
Supplement: Supplementary file 1 — Additional file 1. Supplementary Tables S1-S3 and Figures S1-S5. [file 40246_2021_369_MOESM1_ESM.docx]

**Supplementary Material**

**Supplementary Table S1**. Rationale behind each type of sample collection.

| **Tissue Type** | **Amount of Sample Collected** | **Rationale Behind Sample Collection** |
| --- | --- | --- |
| Blood | 20 mL | Blood represents the mesoderm germ layer. Blood was collected in EDTA tubes, DNA was extracted from leukocytes. Cell free DNA was collected from blood as well, cell free DNA flows through the blood stream. |
| Buccal | A single swab | DNA from buccal cells were extracted. A buccal sample represents the ectoderm germ layer. |
| Urine | 50 mL | DNA was extracted from urethral cells and free-floating DNA in urine. Urine represents the endoderm germ layer. |
| Sputum | 4 mL | DNA was collected from sputum in a saliva kit. Sputum represents the mesoderm and ectoderm germ layers and is similar to whole blood. |
| Hair | Multiple shafts with hair follicles | DNA was extracted from hair follicles. Hair represents the ectoderm germ layer. |
| Nail | Multiple toe nail clippings | DNA was extracted from nail clippings. Nails represent the ectoderm germ layer. |

**Supplementary Table S2.** Phenotypes of 21 probands whose parents tested positive for low-level somatic mosaicism via amplicon NGS.

| **Gene** | **Variant** | **ACMG** | **DD/NDD** | **Proband phenotype** | **Symptoms in Probands Initially Referred for ES testing** |
| --- | --- | --- | --- | --- | --- |
| *CACNA1C* | c.694G>C | VUS | DD | Timothy Syndrome [MIM:601005] | Prenatal history of polyhydramnios |
| *COL6A3* | c.218A>G | VUS | NDD | Bethlem myopathy [MIM:58810] | History of global developmental delays, history of neuroregression post seizures, hypotonia, relative macrocephaly, and failure to thrive. |
| *COL11A1* | c.3816+2dupT | Pathogenic | NDD | Stickler Syndrome 2: [MIM:604841] | Bilateral talipies equinovarus, retinal lattice degeneration, sensourineal hearing loss, and dysmorphic features. |
| *DLL4* | c.586G>A | Likely pathogenic | NDD | Adams-Oliver syndrome [OMIM:616589] | History of IUGR, failure to thrive, idiopathic vein thrombosis, s/p splendectomy,and spleno renal shunt, complicated gastrointestinal bleeds |
| *HUWE1* | c.12257G>A | Likely pathogenic | DD | Mental retardation, X-linked, turner type [MIM:30076] | Delayed motor milestones, delayed speech, autism spectrum disorder behavior problems, intellectual disability, ataxia, lack of coordination, dysmorphic features, sleep disturbances, sleep,apnea |
| *IFT172* | c.4161-1G>A | Pathogenic | NDD | Retinitis pigmentosa [MIM:616394] | Developmental delays, autism spectrum disorder, delayed speech, nystagmus, retinal folds, retinal detachment, strabismus, ablymopia and myopia. |
| *KIF1A* | c.1132C>T | VUS | NDD | Polyhydramnios | Gastrointestinal dysmotility ,delayed motor milestones, visual impairment, static encephalopathy attributed to birth trauma, patient foramen ovale, brain atrophy |
| *KIF1A* | c.757G>A | Pathogenic | DD | Mental retardation, autosomal dominant 9 [MIM:614255] | Polyhydramnios, intrauterine growth restriction, respiratory failure, primary apnea of newborn, pulmonary edema, atelectasis, flexion contractures, congenital multiple arthrogryposis |
| *NRXN2* | c.3382G>A | VUS | DD | Autism spectrum disorder | Global developmental delays, a history of speech regression, aggressive behavior, and macrocephaly |
| *PDCD10* | c.474+5G>A | Pathogenic | NDD | Cerebral cavernous malformations 3 [MIM:603285] | Neutropenia, thrombocytopenia, a history of deep vein thrombosis and brain abscesses |
| *PIGA* | c.986T>C | Likely pathogenic | DD | Multiple congenital anomalies-hypotonia-seizures syndrome 2 [MIM:300868] | Delayed motor milestones, delayed visual maturation, epileptic encephalopathy, deep gray matter diffusion, hypsarrythmia, hypertonia and postnatal failure to thrive |
| *PTEN* | c.542T>C | Pathogenic | NDD | Cowden syndrome 1 [OMIM:158350] | Thoracic aortic aneurism, abnormal tricuspid valve morphology, abnormal aortic arch morphology, myopia, macrocephaly, cognitive impairment, and autistic behavior |
| *PTPN11* | c.923A>G | Pathogenic | DD | Noonan syndrome 1 [MIM:163950] | Short stature, feeding difficulties, mild speech delay, mild dysmorphic features, down slanting palpebral, fissures, and posteriorly rotated ears |
| *PTPN11* | c.923A>G | Pathogenic | DD | Noonan syndrome 1 [MIM:163950] | Atrioventricular septal defect, polyvalvular thickening, bicuspid aortic valve, deep sacral dimple |
| *PTPN11* | c.922 A>G | Pathogenic | DD | Noonan syndrome 1 [MIM:163950] | Connective tissue disorder, delayed speech and language development, delayed motor milestones, developmental regression, seizures, hypertelorism, abnormality of the external nose |
| *RBFOX1* | c.474 + 6T>C | VUS | DD | Autism spectrum disorder | Intractable epilepsy, global developmental delay, intellectual disability, autism, Floating Harbor Syndrome |
| *SMARCA2* | c.2254 G>A | Likely pathogenic | DD | Nicolaides-Baraister syndrome [MIM:601358] | Global developmental delay, developmental regression, delayed cranial suture closure, dry cracking skin, fine hair, failure to thrive, sister has significant history of Asperger’s syndrome and ADHD |
| *SMARCA4* | c.2936G>A | Pathogenic | DD | Coffin Siri syndrome 4 [MIM: 614609] | Congenital diaphragmatic hernia, dextrocardia, pulmonary hypoplasia, polyhydramnios, cleft palate, macrocephaly, dysmorphic features |
| *TPM1* | c.475 G>A | Likely pathogenic | NDD | Cardiomyopathy [MIM:115196] | Cardiac arrest, tricuspid regurgitation, mitral regurgitation |
| *USP7* | c.238A>T | Likely pathogenic | DD | Intellectual disability and autism spectrum disorder [PMID: 26365382] | Motor delays, aortic root dilation, generalized hypotonia abdominal distension, syndactyly and dysmorphic features |
| *ZBTB20* | c.1916G>A | Likely pathogenic | DD | Primrose syndrome [MIM:259050] | Developmental delay, delayed speech, intellectual disability, macrocephaly, possible craniosynostosis |

DD, developmental disorder, NDD, non-developmental disorder.

**Supplementary Table S3**. Comparison of the levels of parental mosaicism taken over two time points in DNA extracted from lymphocytes in the same individual. NA, none accountable. ND, not detectable

| **Parent** | **Gene** | **Variant** | **Year of First Blood Sample** | **Year of Second Blood Sample** | **Percent VAF in First Blood Sample** | **Percent VAF in Second Blood Sample** |
| --- | --- | --- | --- | --- | --- | --- |
| M1 | *IQSEC2* | c.3279G>A | 2012 | 2021 | NA | 14% |
| M1 | *USP7* | c.238A>T | 2018 | 2021 | 11% | 9% |
| M2 | *PTPN11* | c.923A>G | 2019 | 2021 | 0.3% | 0.3% |
| M3 | *PTPN11* | c.923A>G | 2017 | 2021 | 0.3% | 0.3% |
| M4 | *COL11A1* | c.3816+2dupT | 2018 | 2021 | 0.2% | ND |
| M5 | *CACNA1C* | c.694G>C | 2019 | 2021 | 2% | ND |


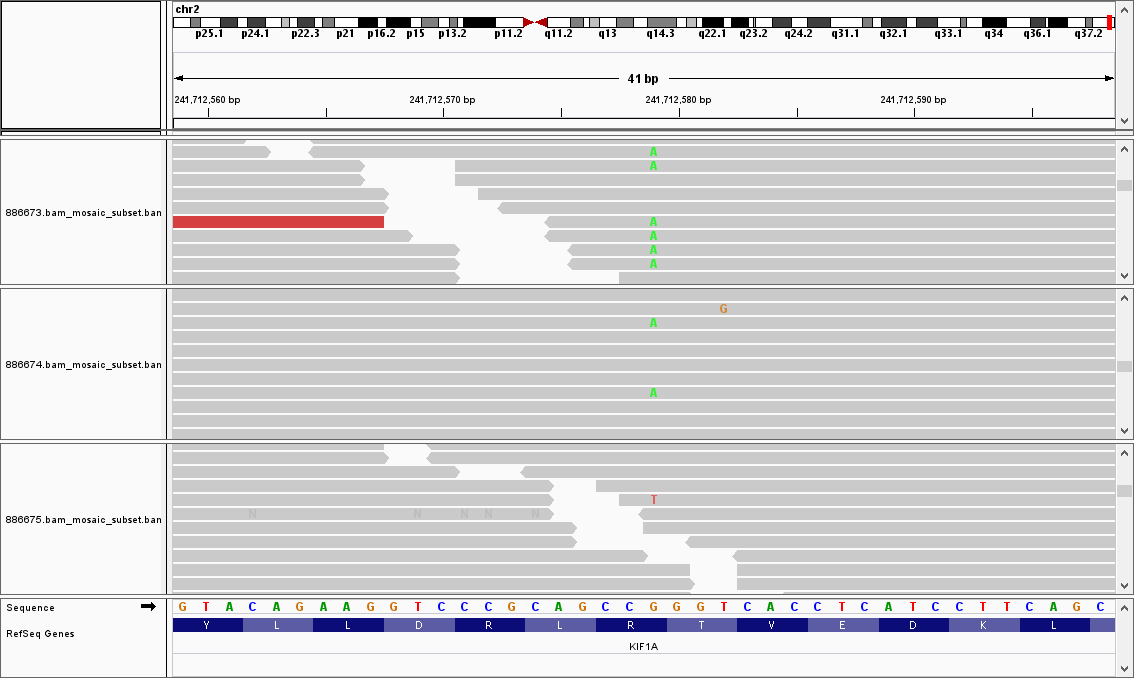


Father

Mother

Proband

**Supplementary Figure S1**. Visualization of the c.757G>A variant in exon 13 of the *KIF1A* gene seen in trio ES BAM files from a mother and the proband. Trios identified automatically via a bioinformatics script that queried the ES database at BG were screened manually for alternate reads at the position of the clinically relevant variant.


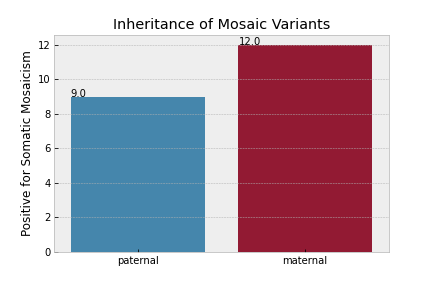


**Supplementary Figure S2.** Parental origin of the 21 mosaic variants identified via amplicon NGS.


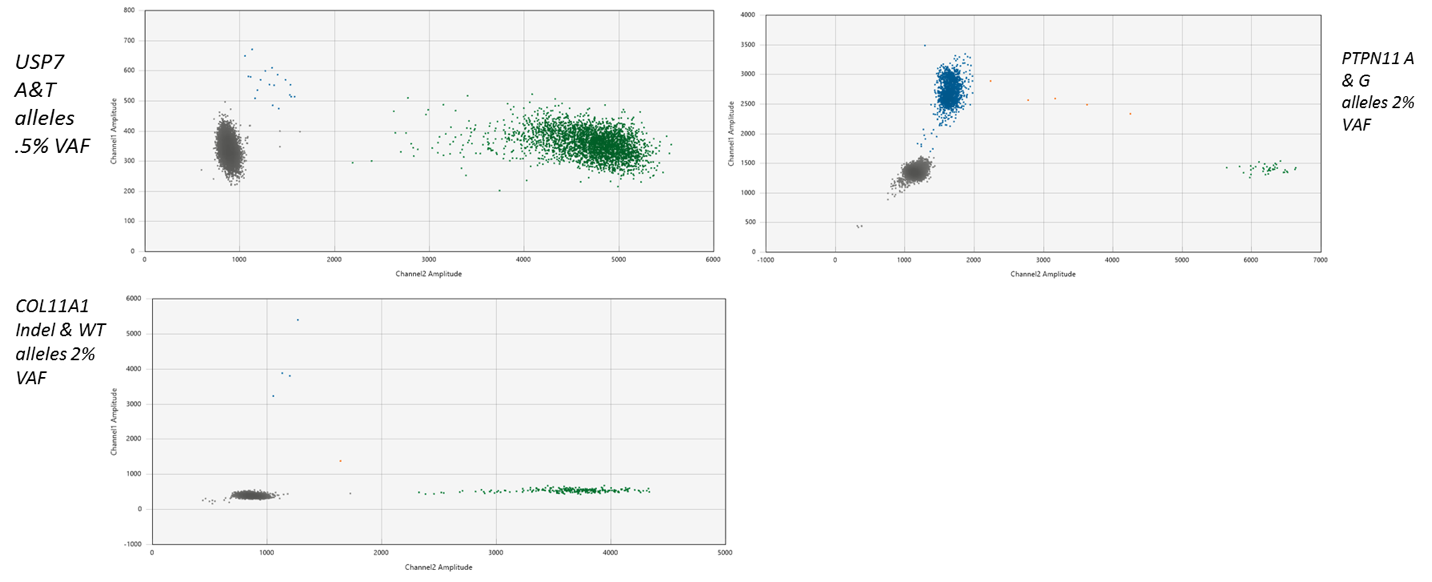


**Supplemental Figure S3.** Results of ddPCR experiments performed to determine the limit of detection (LOD) using gblocks.

***
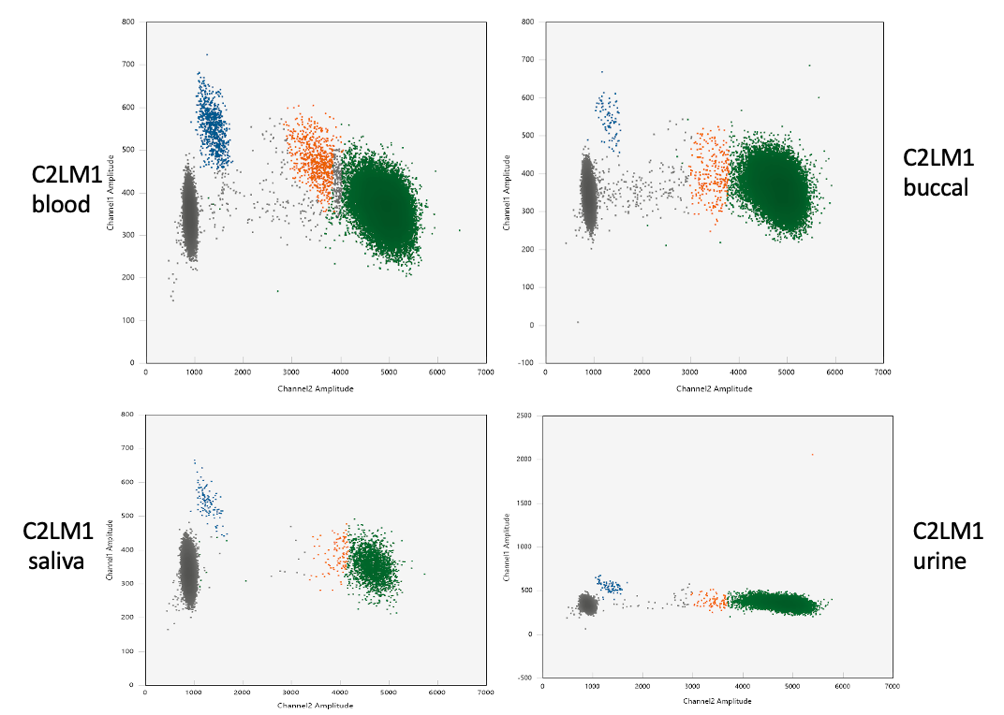
***
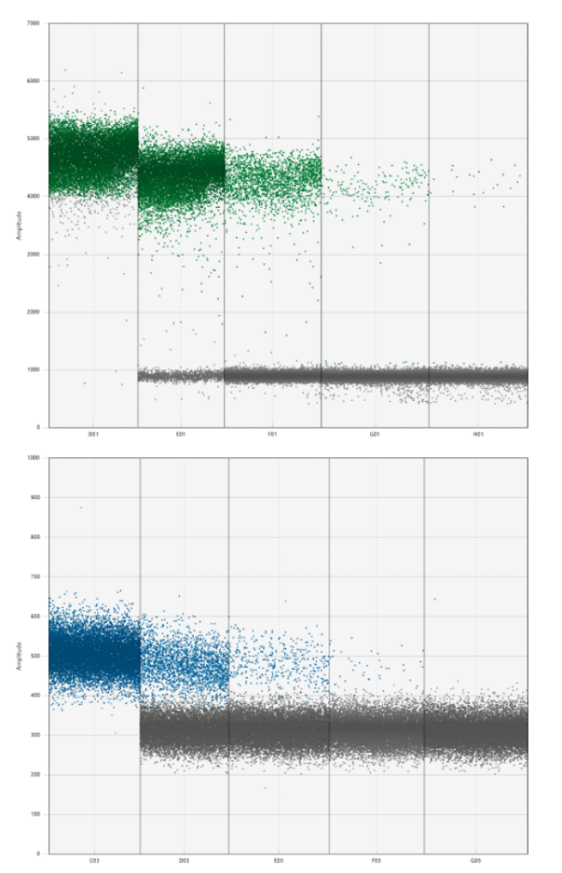

**Supplementary Figure S4**. a) USP7 T allele dilution series (4070 copies/ul to 2.65 copies/ul) in parent M1. b) A allele dilution series (6868 copies/ul to 0.0833 copies/ul). c) ddPCR 2-D plots of droplets of USP7: T and A alleles in different tissues. d) comparison of VAF between NGS and ddPCR across tissue type.

**
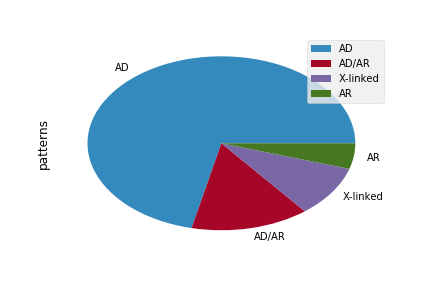
**

**Supplementary Figure S5.** Pie graph showing inheritance pattern of positive mosaic cases.
